# Rational Pharmacotherapy in Infectious Diseases: Issues Related to Drug Residues in Edible Animal Tissues

**DOI:** 10.3390/ani11102878

**Published:** 2021-10-01

**Authors:** Lucila Canton, Carlos Lanusse, Laura Moreno

**Affiliations:** Laboratorio de Farmacología, Centro de Investigación Veterinaria de Tandil (CIVETAN), UNCPBA-CICPBACONICET, Facultad de Ciencias Veterinarias, Tandil CP7000, Argentina; cantonlu@vet.unicen.edu.ar (L.C.); clanusse@vet.unicen.edu.ar (C.L.)

**Keywords:** rational use of veterinary drugs, drug residues in food, drug regulation, maximum residue limits, drug residue monitoring, drug residue effects, impact of cooking

## Abstract

**Simple Summary:**

Drug use is essential to treat diseases in food-producing animals. The most widely used drugs are antiparasitics and antimicrobials. They contribute to guaranteeing good-quality food in sufficient quantity for human consumption. When using veterinary medicines, it is essential to follow the instructions on the package label. Administering the correct dose by the indicated route in the animal species for which the drug is labeled is critical. After a pharmacological treatment is administered to livestock, a period (indicated on the label) must often elapse before the tissues from the treated animals can be consumed by humans. Veterinary drug residues are controlled by taking food samples to verify that drug concentrations do not exceed the permitted limits. This allows authorities to know if the medicine use is correct or if suitable corrective measures should be taken. When label’s directions are not followed, drug residues may appear in food. The residues exceeding the permitted limits established by the authorities can produce unfavorable consequences, mainly on the consumer’s health. The food trade and even the environment can be affected by drug residues in animal tissues. Therefore, the correct use of drugs in livestock is critical, which includes respecting the rules to avoid residues in food for human consumption.

**Abstract:**

Drugs are used in veterinary medicine to prevent or treat animal diseases. When rationally administered to livestock following Good Veterinary Practices (GVP), they greatly contribute to improving the production of food of animal origin. Since humans can be exposed chronically to veterinary drugs through the diet, residues in food are evaluated for effects following chronic exposures. Parameters such as an acceptable daily intake (ADI), the no-observed-adverse-effect level (NOAEL), maximum residue limits (MRLs), and the withdrawal periods (WPs) are determined for each drug used in livestock. Drug residues in food exceeding the MRLs usually appear when failing the GVP application. Different factors related either to the treated animal or to the type of drug administration, and even the type of cooking can affect the level of residues in edible tissues. Residues above the MRLs can have a diverse negative impact, mainly on the consumer’s health, and favor antimicrobial resistance (AMR). Drug residue monitoring programmes are crucial to ensure that prohibited or authorized substances do not exceed MRLs. This comprehensive review article addresses different aspects of drug residues in edible tissues produced as food for human consumption and provides relevant information contributing to rational pharmacotherapy in food-producing animals.

## 1. Introduction

Infections account for some of the most significant diseases worldwide, both in animals and humans, and are of enormous socioeconomic importance. Moreover, the appearance of new diseases should always be kept in mind [1]. The COVID-19 pandemic is the most recent infectious disease outbreak to emerge at the human–animal–environment interface, but it is not the first time an unknown or new virus has developed from close contact between humans and wildlife [2]. Viral, fungal, parasitic, or bacterial-mediated infectious diseases remain a significant threat to animal production and cause large deficits to the livestock economy. Therefore, effective control is essential for the profitability of intensive livestock production [3,4]. In addition, the growing world population increasingly demands affordable sources of protein from food animals and animal products, requiring improvements in livestock health care [5]. In this context, it is evident that animal food supply will continue to rely on the use of drug-based therapies.

Veterinary drugs are chemical agents used to prevent or treat animal diseases. Rationally administered to food-producing animals, following Good Veterinary Practices (GVP), they favor the production of abundant food, such as meat, milk, eggs, and honey. In contrast, several adverse effects, such as drug residues in food exceeding safe levels for humans, may appear when GVP standards are not met. Consequently, one of the essential principles prescribed by the international legislation is that foodstuffs obtained from animals treated with veterinary drugs must not contain levels of residues either of the parent molecule or its metabolites that pose a risk to the consumer’s health. 

Focusing on drug residues in food-producing animals, antibacterial and antiparasitic compounds are among the chemicals with the most outstanding involvement in animal production due to the magnitude of their use. In fact, because of the high economic impact and the direct repercussion on animal health, antiparasitic drugs currently represent, after biologicals, the second-largest position (23% of market share) in the world animal health market. Antibiotics rank third with 16% of sales [5]. The pharmacotoxicology and the patterns of tissue residues of different drugs used in livestock animals, which are beyond the scope of the current work, have been widely described in the literature [6,7,8]. To integrally assess the topic, this comprehensive review article addresses different aspects of drug residues in edible tissues produced as food for human consumption and provides relevant information contributing to rational pharmacotherapy in food-producing animals.

## 2. Issues on the Regulation of Drug Residues in Food of Animal Origin

Many chemicals are of great concern to both human and environmental health due to their acute or chronic toxicity with extensive exposure. Animal or plant foods are one of the primary sources of human exposure to these unwanted chemicals. In the food supply chain, there are many steps in which chemicals can come into contact with food, from primary production to final consumption, and this has to be controlled to assess the safety of food products [9]. In this matter, essential principles have been introduced, such as risk analysis, traceability, and the "One Health" paradigm, in which a multisectoral, interdisciplinary, and collaborative approach is crucial for attaining optimal health for animals, the environment, and humans. This, in turn, implies an integrated food chain "from farm to fork" principle recently reaffirmed (Farm to Fork Strategy) as the heart of the European Green Deal [10]. For the regulation of veterinary drug residues in food of animal origin, this three-step risk analysis procedure (risk assessment, risk management, and risk communication) is also applied to guarantee consumer health. Following this procedure, but using different approaches, diverse regulatory organizations at the international level (Joint FAO/WHO Expert Committee on Food Additives (JECFA); The Food and Drug Administration (FDA); The European Medicines Agency (EMA)) work to provide scientific information that allows the control of drug residues in food. Although these methodologies are not described in detail here, some particular relevant issues are briefly discussed. 

### 2.1. How Veterinary Drug Residues Are Regulated

In general, veterinary drugs and drugs for humans are the same, but the way in which they are regulated differs, especially for drugs used in food production animals. Since humans can be exposed chronically to veterinary residues through the diet, veterinary drug residues in food are evaluated for effects following chronic exposures, so an acceptable daily intake (ADI) must be established. For this purpose, various toxicity studies are carried out in laboratory animals. Sometimes, human data, in vitro, and in silico studies are also considered. From these repeated-dose exposure studies in experimental animals, a point of departure (POD) is most often established, e.g., the no-observed-adverse-effect level (NOAEL). The ADI is obtained by dividing the NOAEL by a factor (100), providing a human health-based guidance value (HBGV) for chronic or long-term exposures to residues in food [11]. After the ADI is established, the maximum residue limits (MRLs) for the individual food commodities are determined. These are defined as “The maximum concentration of residue resulting from the use of a veterinary drug (expressed in mg/kg or μg/kg on a fresh weight basis) that is recommended to be legally permitted or recognized as acceptable in or on a food” [12]. For the US FDA, the equivalent parameter is named tolerance, and it is specific for each drug and tissue. Moreover, regarding the marketing authorizations for veterinary medicinal products, it is necessary to determine withdrawal periods (WPs). The MRLs or tolerances assigned are taken into account for this purpose. The WP is the time after the last administration of the veterinary medicinal product during which the animal must not be slaughtered or during which milk or eggs must not be intended for human consumption, ensuring that residues will not exceed the MRLs. The WP is based on residue studies conducted under the labeled conditions of use (type of animal, dosage, route of administration) to ensure that residues above tolerance/MRL will not be present in animal products used as human food. The WP enables the animal to metabolically reduce the drug level in tissues to levels that are not of public health concern. Toxicological evaluations and parameters (NOAEL, IDA, MRL, tolerances) established for veterinary drugs are reported by agencies or official bodies, some of which are shown in Table 1 with their links. 

### 2.2. Acute Reference Dose Instead of Acceptable Daily Intake

An aspect to be considered is that the level of veterinary drug residues in a single meal can cause adverse effects in humans, such as the acute intoxication by clenbuterol residues in the liver or meat [13,14,15,16]. Of particular interest are the residues in meat that may appear at the injection site (IS) after the administration of injectable formulations [17]. When drug residues in the IS are much higher than in other edible tissues, it constitutes a challenge for risk assessment for the consumer’s health. The administration of injectable formulations to food-producing animals may result in acute toxicity after consuming the entire injection site with high drug residues in a single meal [18,19]. It is also possible to consume a piece of meat/tissue where the veterinary drug residue is concentrated. In such cases, the ADI may not be the most appropriate value for quantifying the level above which a single exposure (after a single meal or during one day) can produce adverse effects.

In contrast, establishing an HBGV based on acute effects, such as the Acute Reference Doses (ARfD), will be better to address this concern. The ARfD approach has been developed to provide an HBGV for chemicals that can cause adverse effects following acute or short-term exposures in humans consuming food containing residues. It is surprising that although several guidelines were published for calculating the ARfD [20,21,22,23], none of them specifically reported on veterinary drug residues. Fortunately, given the importance of the topic, some guidance for establishing ARfD for veterinary drug residues in food was later published [11,24] and applied for several drugs [25]. 

### 2.3. Metabolite to Parent Drug Ratio

Another critical issue is using a fixed main metabolite to parent drug (M/D) ratio to establish drug MRL/tolerances and withdrawal times. These ratios are determined in specific conditions, such as (a) early studies for limited early time points in a small number of animals; (b) studies carried out in healthy animals when the drug is used later in sick animals; (c) studies where the specific formulation is not administered. Consequently, frequent drug residue violations in food-producing animals may occur even though the animals are slaughtered according to regulatory-labeled withdrawal times [26,27,28]. The M/D ratio is altered substantially by diseases because of changes in drug metabolism, depending on the disease severity. A three-fold change in hepatic metabolic rate was estimated in diseased animals [28].

### 2.4. Lack of Global Harmonization: MRLs/Tolerances and Withdrawal Periods

**Figure 1:**
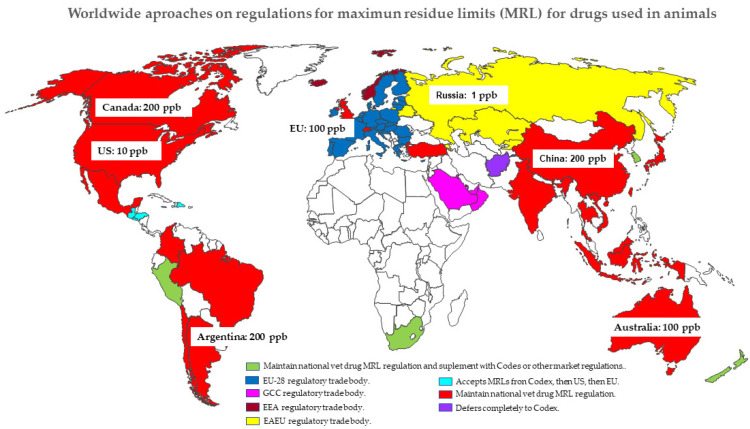
Countries´ classification according to the approaches taken for the regulation of maximum residue limits in food (MRLs/Tolerances) for veterinary drugs. The MRLs for oxytetracycline in cattle muscle adopted in some countries are shown as an example. GCC: Gulf Cooperation Council. EAEU: Eurasian Economic Union. EEA: European Economic Area. Data adapted from Stevenson, 2017 [29].

The procedures used for the regulation of drug residues in foods of animal origin differ between countries. Different philosophies and methods are applied to determine MRLs or tolerances and WPs for drug residues in edible tissues. MRL values are crucial when importing or exporting animal products. The fact that the regulations are not standard worldwide, as shown in Figure 1, leads to barriers to trade. Food producers need to know how veterinary drug MRLs are regulated in importing countries. Accordingly, they are included in one of four different approaches: 1. Countries that maintain national veterinary drug MRL regulations. 2. Countries that maintain national veterinary drug MRL regulations but supplement with Codex or other market regulations. 3. Countries that belong to some regulatory trade body. 4. Countries that defer to Codex or other markets’ veterinary drug MRL regulations [29]. In addition, different approaches, many based on statistics, have been used to determine the withdrawal time for veterinary drugs in food-producing animals. These include the use of half-life multipliers (number of half-lives contained within the withdrawal time), the withdrawal-period estimator algorithm [30,31], the non-parametric methods for specific drugs [32], and the physiologically based pharmacokinetic modeling [33,34,35,36]. The statistical linear regression method is the most widely used and recommended by both FDA (at 99% of tolerance interval with 95% confidence intervals) and EU (at 95% tolerance with 95% confidence intervals), although it was not considered the best approach [37]. These singularities, related to the different MRLs, procedures for calculating WP, and drug tissue residue data considered, have been demonstrated. When Udiani et al. [38] estimated the WP for veterinary drugs, such as sulfamethazine in swine liver [35] and flunixin in calf liver [26], different values were obtained depending on the data and the MRL taken into account. In the same way, tissue residue levels of florfenicol and tylosin in different tissues were considered to determine the WP by the linear regression method with both recommendations (FDA and EU), and different values were obtained [39]. Consequently, this lack of harmonization of MRLs and WPs could lead to obstacles to food trade between countries [40].

### 2.5. Extra-Label Use: When Can It Be Allowed?

It is essential to know that the extra-label use of different drugs is allowed in “particular situations”. It means that a medicinal product can be exceptionally intended for non-authorized use in some particular cases, for example, to avoid unacceptable suffering to the animals involved. In such cases, the administration of a non-authorized medicinal product to an animal or a small number of animals on a particular holding can be permitted under a veterinarian’s responsibility. In any case, it should be noted that this practice is well regulated [41,42,43,44,45] to prevent the appearance of illegal residues in human food, such as meat, eggs, milk, or other products. Consequently, the procedures for proper extra-label use of drugs in food animals are much more restrictive than those for companion animals [46]. In the USA, a guide to establishing a withdrawal period under the Animal Medicinal Drug Use Clarification Act 4 [47] has been published [48]. Accordingly, veterinarians can use drugs in food-production animals in an extra-label manner, provided that an appropriately extended withdrawal time is established [31]. For example, tylosin is the only macrolide approved for use in poultry, but licensed veterinarians can use other macrolides extra-label if they are responsible for ensuring the safety of drug residues in tissues [7]. In the same way, since relatively few antiparasitic drugs are FDA-approved for goats, veterinarians often use antiparasitic drugs approved for sheep in goats in an extra-label manner [49]. A similar situation occurs in the treatment of parasitic diseases in fish farming, for which the approved drugs are scarce. Praziquantel is an anthelmintic available for humans and several animal species, but not for fish. Praziquantel has high efficacy against cestodes and trematodes in fish farming and can be used extra-label under UMDUCA in the USA [50]. It implies that the prescribing veterinarian is responsible for establishing a substantially extended withdrawal period supported by appropriate scientific information. This information can be obtained from such sources as scientific literature, academia, or the Food Animal Residue Avoidance Databank (FARAD) (http://www.farad.org/). Insisting on the rational use of veterinary drugs, veterinarians of animals intended for human consumption must know the regulations governing both the use of approved and extra-label drugs. They must keep in mind if an approved drug for livestock is indicated for the illness being treated. If such a drug exists, the use of extra-label drugs is not appropriate [51].

## 3. Negative Impact of the Presence of Residues on Edible Animal Tissues 

**Figure 2:**
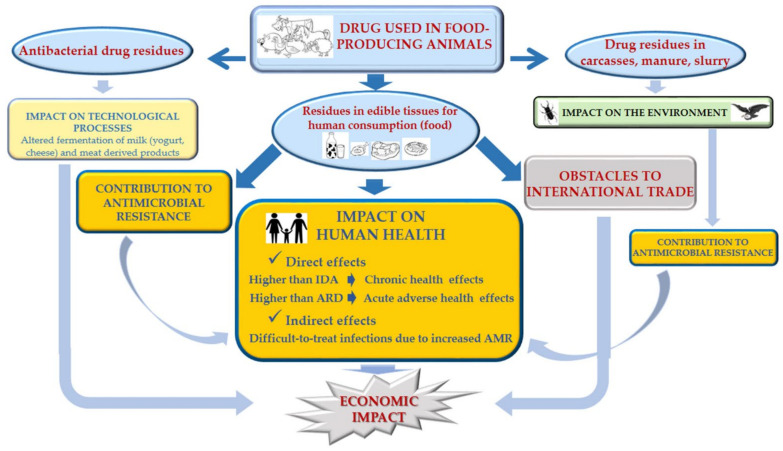
Schematic representation of the main adverse consequences of the presence of drug residues in edible animal tissues.

When veterinary drugs are administered to food-producing animals, rules must be respected to avoid residue levels in the food obtained exceeding the maximum permitted values. Otherwise, as schematically shown in Figure 2, these residue levels could produce various unwanted effects, some of which are described below.

### 3.1. Economic Impact

The overall consequences of drug residues in food are economic, because international trade is blocked, altering the technological processes that require microbial fermentation in the food industry (for antibiotic residues), and human health effects. Health problems are discussed below, but the most relevant concern is antimicrobial resistance, leading to a higher frequency of prolonged hospitalization, long illness, and increased mortality [52,53]. At the level of international trade, when the MRLs for veterinary drugs set by the importing country are not met, a violation may be evidenced, which would lead to problems for the sender, the exporter, or the industry. The severity of the violation depends on the importing market and the level of the drug residue detected. Animal products can be detained, destroyed, or marked for further testing in the future. Severe or repeated infractions can result in the temporary closure of the commercial relationship with a country [29].

### 3.2. Human Health Impact

Drug residues in food have been related to direct effects on human health. There is no risk of acute toxic effects for most veterinary drugs after low levels of residues in food are ingested. However, as described in safety testing toxicity studies (required before its use authorization in the market), they were demonstrated to cause some harmful health effects after chronic ingestion of low levels of residues in food. Then, long-term chronic studies identify whether diseases such as cancer, reproductive disorders, mutagenicity, are associated with a particular drug. In addition, some epidemiological studies reported undesirable effects associated with chronic drug ingestion in food. The long exposure of the mother to antibiotics in food or drinking water has been associated with vertical transmission and increased risk of childhood obesity [54,55,56,57].

Although there are not many recent cases, antibiotic residues in meat have been reported to cause toxic or allergic reactions in humans. The prevalence of sensitivity to antibiotics varies but it has been estimated to affect around 7% of the population. However, not all of these people will have an allergy to antibiotic residues since the food levels are usually below the threshold that would induce it [58,59]. Concentrations of residual veterinary drugs in foods are not high enough to cause an initial hypersensitive reaction, causing the effect when the person was previously sensitized to the drug [60]. Allergic reactions may involve skin rashes or asthma and, in the worst cases, anaphylactic shock. Penicillin allergic reactions are the most frequent, affecting up to 10% of people receiving these drugs therapeutically. Anaphylactic reactions were observed after consumption of beef or pork containing penicillin [58,61]. Sulphonamides may cause allergic reactions in up to 3% of those using these drugs. 

Chloramphenicol caused fatal aplastic anemia, which results in death in approximately 70% of the cases, and people recovering have high chances of experiencing acute leukemia [62]. Beta-agonist drugs, such as clenbuterol, are used therapeutically in animal medicine for specific effects on smooth muscle, such as a bronchodilator. When misused at higher doses, they can also act as growth promoters by stimulating an increase in muscle mass and a reduction in adipose tissue. It is known that, in most countries, the use of beta-agonists in food-producing animals is prohibited except for well-defined therapeutic purposes and under strict veterinary control. As mentioned above, numerous cases of acute poisoning with clenbuterol residues in food have been described since the 1990s [13,14,15,16,63,64]. Surprisingly, although it is a prohibited and controlled substance, headlines of acute clenbuterol poisoning keep appearing [65].

### 3.3. Environmental Health Impact

As regards “the one health” paradigm, it is important to note how the use of drugs in food-production animals and drug residues in tissues could directly affect wildlife and the environment. The unintentional arrival of veterinary drug residues through the carcasses of treated production animals can threaten wildlife. Drug residue levels have been detected in wild animals that were never treated. 

Topical antiparasitic drugs used in livestock (diazinon and permethrin) were quantified in bearded vultures [66]. These likely represented accidental exposure due to the legal use of these veterinary pharmaceuticals. Such topical antiparasitic used in livestock could be related to the mortality and breeding impairment in this endangered species. A high percentage of samples (29%) taken from carrion disposed of for feeding endangered scavenger birds had antibiotic residues. Oxytetracycline (at the highest concentration: 1452.68 ng/g) and trimethoprim residues were the most common, with higher frequency in goats (42.9%) than in sheep (24.2%) [67]. Fluoroquinolones (marbofloxacin, enrofloxacin, and its metabolite ciprofloxacin) and a non-targeted β-lactam (nafcillin) were detected in vulture plasma. Low plasma concentrations (up to 20 ng/g of enrofloxacin and ∼150 ng/g of marbofloxacin) were quantified in a high proportion of individuals (92%) in different colonies and on different dates, suggesting potential ingestion throughout nestling development [68]. Similarly, fluoroquinolones, mainly enrofloxacin plasma concentration (54.5 ± 6.6 ng/mL), were found in the nestlings of the golden eagle (Aquila chrysaetos) [69]. Disposal of diseased and medicated livestock carcasses at feeding stations may imply the unintended availability of veterinary pharmaceuticals and pathogens in the feed of threatened wildlife [68]. This poor management of the livestock carcasses can lead to antibiotic resistance in the scavengers that ingest them, such as vultures [69,70,71]. Therefore, this practice should be regulated to minimize the risk in scavenger birds [8].

Furthermore, the veterinary use of the non-steroidal anti-inflammatory drug (NSAID) diclofenac in domestic ungulates was the leading cause of the decline in the vulture population in India [72,73,74,75]. When the effects of diclofenac were experimentally studied in various breeds of vultures, death occurred within a few days with extensive visceral gout and kidney damage [72,76,77]. The pathology was similar to that found in most vulture carcasses collected from the wild [72,73,74]. Therefore, several Asian countries (India, Pakistan, and Nepal) took action in 2006 to prevent the veterinary use of diclofenac on livestock, the source of vulture contamination. Although the vulture population has improved due to a decrease in diclofenac use in livestock, further efforts to remove diclofenac from vulture food are still needed to complete the recovery of the species [78]. This problem is highly relevant, especially when it can be extended to other countries.

In Spain (and Europe), the first case of diclofenac poisoning in cinereous vultures has been recently reported. In September 2020, a vulture chick was found dead in the nest. The autopsy revealed severe generalized joint and visceral gout, and diclofenac concentrations were detected at 26.5 and 51.4 ng/g in the liver and kidney, respectively [79].

The worldwide extensively used macrocyclic lactone ivermectin is poorly metabolized and excreted (more than 90%) actively in the feces of treated livestock (69% in sheep, 43% in pigs, 39% in cattle) [80,81,82]. Consequently, it can enter the environment directly when the animals are grazing or indirectly through the application of manure and slurry on the land [83]. Numerous studies have addressed the potential effect of ivermectin on non-target organisms [80,84,85,86,87]. A negative effect of ivermectin has been shown both on terrestrial (especially dung beetles) and aquatic (being *Daphnia Magna* the most sensitive) invertebrates, fish [88], and even plants, in which significant inhibition of germination was observed [89,90].

## 4. Relevance of Monitoring Drug Residue Programmes

The monitoring of drug residues in food of animal origin is useful to ensure that prohibited substances (due to their toxicity) are not being used and that authorized substances do not exceed maximum residue levels by complying with the withdrawal times between treatment and slaughter [91].

### 4.1. Official Monitoring Drug Residue Programmes

Residue monitoring consists of a sampling of foodstuffs to determine the trends in the use of veterinary drugs, and accordingly, further and directed monitoring can be carried out. In this sense, some countries have implemented well-organized and regulated surveillance programmes (EU under Directive 96/23/E.C.; Canada through the National Chemical Residue Monitoring Program, or NCRMP; the USA through the National Residue Program of the Food Safety and Inspection Service; and New Zealand under the Food Residues Surveillance Programme). In other countries with different socioeconomic realities, strict programmes to control drug residues in food can be more difficult to implement. In general, drug residue monitoring in animal-derived foods takes place annually. Specifically, the EU member countries monitor and report on these residues every year. 

The checked molecules are grouped into six categories: hormones, beta-antagonists, prohibited substances, antibacterials, other veterinary drugs (anthelmintics, anticoccidials, carbamates, and pyrethroids, sedatives, NSAIDs, and corticosteroids), and other substances/environmental contaminants. Samples of bovines, pigs, sheep and goats, horses, poultry, rabbits, farmed game, and other animals are monitored. In 2021, the EFSA published the latest report about residues found during 2019. Fortunately, a low incidence of samples with drug residues above the maximum permitted levels (non-compliant samples) was observed, 1191 (0.32%) out of the 368,594 targeted samples. Only 0.14% of the samples exceeded the allowed level for antibacterials, with the highest frequency found in honey. The highest percentage of non-compliant samples was for the NSAIDs, which was low (0.19%) [92]. For antiparasitic drugs, low incidence of anthelmintic non-compliant samples was reported in bovines (0.05%), poultry (0.02%), sheep and goats (0.48%), pigs (0.09%), horses (0.8%), and milk (0.08%). Anticoccidial non-compliant samples were found in pigs (0.04%), poultry (0.03%), sheep and goats (0.09%), and eggs (0.21%). 

### 4.2. Unofficial Drug Residue Studies: From Residue Quantification and Exposure Assessment to Detailed Risk Characterization

Following different approaches, numerous research studies have been carried out to quantify drug residues in animal-derived food to evaluate their risk to human health. Some authors [93,94] determined antimicrobial residues in meat samples from several animal species and eggs using bacterial growth inhibition tests. Based on quantified residue levels in edible tissues, they concluded that there was a high risk of public exposure to antimicrobial residues through the consumption of animal tissues. In the same way, other researchers analyzed pork meat, liver, and kidney samples for tetracyclines, fluoroquinolones, sulphonamides, chloramphenicol, and β-agonists residues. However, they concluded that the health risk due to chemical hazards in pork did not seem serious [95].

To assess the risk of drug residues to human health after consumption of food of animal origin, it is necessary to estimate the potential exposure to veterinary drug residues in food. In generic terms, calculation of dietary exposure could be expressed as follows: [96]

Dietary exposure = Concentration (µg/g) * Edible tissue consumption (g) / Body weight (g)

where *Concentration* refers to the concentration of drug residues quantified in the specific food; *Edible tissue consumption* corresponds to the daily amount of this edible tissue consumed; *Body weight* (g) indicates the consumer’s body weight.

In general, the daily drug exposure value, represented by the *estimated daily intake* (EDI), is compared with the drug’s *admissible daily intake* (ADI), which is the international toxicological reference value recommended for chronic exposure. The calculation of the exposure, more precisely the "dietary exposure assessment", obtained by applying the equation mentioned above, could be made using different approaches. The simplest one is the "deterministic or point estimate". This approach uses single values for concentration and food consumption, which could be the drug residue and tissue consumption mean values. A more thorough approach is the "refined deterministic estimate". This approach adopts a statistic distribution for one parameter and a single value for the other. Finally, the "probabilistic or stochastic estimation" uses the statistic (parametric or non-parametric techniques) to generate distributions of the parameters and identify the variables with the greatest influence on the drug residue concentrations in food and food consumption. Consequently, the dietary exposure assessment is made by a distribution of exposure instead of a point value [97].

Several authors carried out exposure assessments for different veterinary residues by calculating the EDI (point estimate or deterministic model). Anthelmintic and antibiotic residues from chicken, fish tissues, and eggs were analyzed by HPLC [98,99,100,101,102]. Some researchers analyzed (by UPLC-MS) beef samples searching for anthelmintic drugs [103]. In none of these studies were the EDI values higher than the established ADIs, suggesting that dietary exposure assessments of all drugs analyzed were within the safe regulatory limits. In contrast, other authors evaluated beef and chicken tissues for antibiotic residues using UPLC-MS, and they determined that consumers were at risk of exposure to some of the molecules studied, which could be injurious to health and wellbeing [104,105].

To our knowledge, there are few reports on drug residue exposure assessments in animal-derived foods using stochastic and probabilistic models. Wang et al. [106] screened tetracycline, fluoroquinolone, macrolide, ß-lactam, sulfonamide, and phenicol residues in meat (pork and poultry) and fresh aquatic products using LC-MS. Canton et al. [107] analyzed fipronil residues in eggs by UPLC-MS. In both studies, EDIs were assessed by Monte Carlo Simulation (using the @Risk software), and the authors concluded that there was an unacceptable risk associated with animal tissue consumption with veterinary drug residues for all the age groups studied. 

Overall, according to data on exposure assessment of veterinary drug residues on different tissues, most of the residues present in animal tissues did not put consumer health at risk. However, the authors pointed out that residual levels were found in most tissues, some of them exceeding the MRLs. Therefore, it must be considered that the evaluated residue could be found in other food ingested by the consumers, and this could change the result of the final exposure assessment. Since available data on veterinary drug exposure assessments by the probabilistic approach are very scarce, it would be necessary to conduct more research on this subject for more complete studies. 

### 4.3. Residues in Food above the Acceptable Levels: Possible Causes

The results of monitoring programmes may not be so encouraging in developing countries. A significant portion of food-producing animals is kept on small and medium-sized individually owned farms whose practices are more difficult to monitor than the well-standardized operations of factory farms [108]. Consequently, recent monitoring studies checking veterinary drug residues in animal-derived food showed a high incidence of residues, mainly antibiotics, over the allowed level [109,110,111,112,113,114]. Overall, such a high occurrence of residual drug violations indicates massive institutional failures in managing veterinary drugs and the surveillance of animal food products [108]. Failure to adhere to recommended withdrawal periods was reported to be the leading cause of non-compliant levels of veterinary drugs in food [115]. The extra-label use of drugs in food-producing animals is a significant public health concern and a contributing factor in illegal residues in edible animal tissues. Improper use of veterinary medicine in food-producing animals by veterinarians and non-veterinarians (e.g., livestock and poultry producers, herders, dealers, haulers) is illegal. This illegal use involves ignoring labeled WP, using the product in a species not listed on the label, using the drug to treat a condition not indicated, administering the drug at a different dosage than stated, or otherwise failing to follow label directions for use and administration of the drug [6].

On the other hand, several factors such as human error, production practices, and management procedures can contaminate non-medicated feed with veterinary drugs. Medicated and non-medicated feeds are usually processed in the same product lines. Consequently, after manufacturing medicated feeds, traces of the drug’s active ingredient will be found in the subsequently processed feeds, an unintentional transfer known as carryover [116,117,118]. The carryover grade depends on the medicated feed amount retained along the production line, the particle size and density, and the electrostatic properties of the premix [6]. When feed intended for the finishing period of food-producing animals is affected by carryover, maximum residue limits in animal food may be exceeded [119]. Moreover, in countries where antibiotics are still allowed as growth promoters, administration in medicated feed is very common [120], and the probability of carryover is higher. Brazil has a high incidence of notifications of veterinary drug residues in the animal products exported [121]. When carryover was evaluated in this country, only 1 out of 25 analyzed lines showed no contamination with other active ingredients [119].

Consequently, animal feeds are controlled, and allowed limits have been established. In the EU, the levels in non-target feed were limited to 1% or 3% of the maximum concentrations allowed in feed [122]. Various practices have been proposed to prevent this contamination: carrying out a sufficient number of rinse batches; respecting production order, producing the drug-free feed before the drug-containing feed; using modern feed manufacturing equipment with less “dead space” for accumulation of medicated feed. Food samples with residues above the allowed level were found due to carryover in the feed, consequently rinsing the feed containers to remove all the contaminated feed at the farm was recommended [6]. However, when the risk of coccidiostat and histomonostat carryover for animal and consumer health was evaluated, it was considered negligible [123,124,125,126,127,128,129,130,131,132]. For antimicrobial drug carryover in feed, the concern is once again the development of resistance. The intestinal microbiota of food-producing animals could be exposed to sub-therapeutic concentrations of antibiotics [6], with consequences for human health as described below in this article.

## 5. Factors Affecting the Drug Tissue Residue Profiles

### 5.1. Drug Treatment-Related Factors 

Aspects related to drug administration, such as the type of formulation, the site and route of administration, the dose, and the time after administration, can influence the pharmacokinetics and tissue drug residues in edible tissues. Veterinary medicines are administered mainly by intramuscular (IM), subcutaneous (SC), and oral routes to food-producing animals. The SC route conferred a higher drug bioavailability in sheep, cattle, and goats when compared to oral or topical administration [133,134]. Similarly, after ivermectin administration to goats, tissue residues were much lower after oral administration than after SC administration [135]. For injectable formulations, the systemic availability and consequent tissue residues depend on the route (IM or SC) and the site of administration [62]. 

The physicochemical properties of the drug, the formulation pH, the local blood and lymphatic circulation, tissue composition, and muscular contractions can influence drug absorption from injection sites. After IM or SC administration, the injection site constitutes a depot, whose progress depends on the properties of the formulation (especially the injection vehicle), the injected volume, the movement of the tissues, and the muscular contraction. In meat production, the SC route is preferred to minimize commercial losses due to injection site reactions and drug residues in the muscle. In this respect, creative methods have been proposed to solve these problems, such as injecting the formulations into the animal’s ear to reduce drug residues in the muscle of meat-producing animals [136]. Indeed, a controversial point of parenteral depot formulations for food-producing animals is the need to administer enough drug to elicit a desired therapeutic effect with the minimum amount of tissue residues, and thus, short or no WP is necessary.

Consequently, intravenous administration (IV) is the only route recommended in food-producing animals for certain drugs in some countries. In the US, flunixin meglumine is the only NSAID labeled to treat pyrexia or inflammation associated with several infectious diseases in beef and dairy cattle [51]. The flunixin meglumine administration following the recommended dose (1.1 to 2.2 mg/kg) and route (IV) in cattle has WPs of 36 hours and four days for milk and meat. Flunixin meglumine is highly irritating when injected IM [137]. It was also associated with the vehicle propylene glycol used in the formulation, known to be irritating to muscle tissue [138].

For this reason, FARAD previously recommended a 30-day slaughter WP for flunixin meglumine products given IM. Moreover, the WP may need to be extended as far as 60 days if multiple doses are administered. IM multiple injections would increase the amount of damaged/necrotic tissue, which would create a drug depot of the drug, prolonging absorption into the circulation, thus creating a flip-flop phenomenon with a highly prolonged absorption phase [51]. This is a desirable phenomenon from a therapeutic point of view, but it is associated with the persistence of tissue residues. The duration of the pharmacological effect will be controlled by dosage drug release and not by the disposition kinetics of the drug. Therefore, the formulation type, classical or long-acting, generic or reference, will influence the residue profile in edible tissues. Conventional formulations of oxytetracycline typically have a labeled WP of 18 to 19 days, whereas long-acting formulations have a 28-day WP. Benzathine penicillin has a labeled WP of 30 days, whereas procaine penicillin has a WP of 10 days [115]. Different generic ivermectin formulations are used in cattle, and significant differences were observed in the plasma concentration profiles. Therefore, different residue tissue levels could be expected [139]. 

To extend the plasma-intestine recycling time of macrocyclic lactone molecules in the host, drug transport modulation has been investigated to delay the drug biliary/intestinal secretions [140,141,142]. The efflux-transport protein P-gp (or other drug transporters) could be involved in both the pharmacokinetic disposition and drug residue profiles. In this way, the study of the potential side effects and changes in the pattern of tissue residues induced by the P-gp modulating agents was recommended [140]. 

In the rational use of veterinary drugs, all the links related to this use—the pharmaceutical industry, wholesale and retail suppliers, veterinarians, farmers—have a role to play. For example, the pharmaceutical industry faces the challenge of developing new formulations for obtaining products with minimized costs, both for the product and for the handling and dosage of the animals, while exhibiting superior efficacy and minimal residues in the tissues intended for human consumption [143].

### 5.2. Animal Host-Related Factors

The differences in the tissue residue profiles between animal species after drug administration are apparent; therefore, veterinary medicines are sold according to previous studies (pharmacokinetics, metabolism, residue profile, and withdrawal periods) for each species. However, particular situations such as that occurring with goats, for which there are very few anthelmintic formulations, cause various problems. Many producers use anthelmintics registered for other species (generally sheep) in goats, which may increase the selection pressure for anthelmintic resistance or affect the safety of drug residues in meat or milk from treated goats [144].

On the other hand, some studies focusing on a single species show that breed, animal age, sex, and body condition influenced drug plasma exposure. These factors could be associated with changes in tissue residue profiles from treated farm animals and, consequently, could influence the estimation of a safe WP. In this line, much work has been done with macrocyclic lactones. Breed influenced plasma drug levels and tissue residues when ivermectin and moxidectin were administered to goats and calves [145,146,147]. Ivermectin residues were more persistent in male than female goats [148].

**Figure 3:**
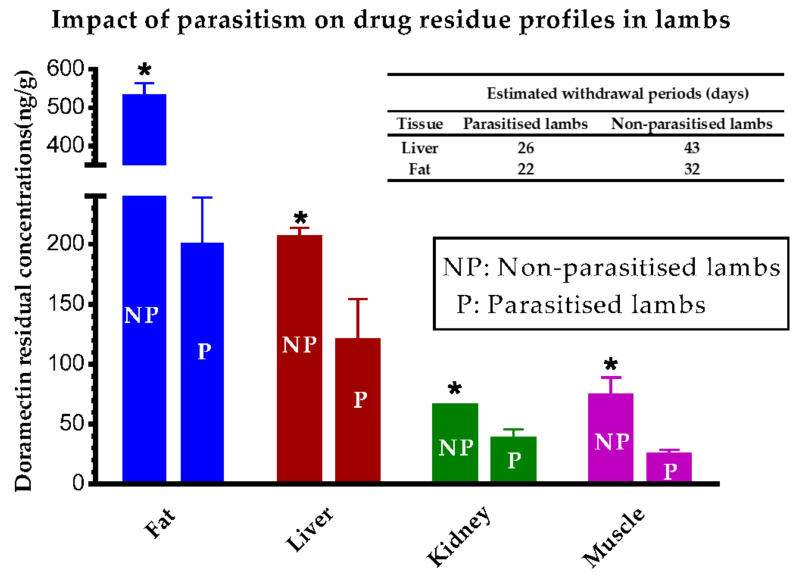
Mean (± SEM) doramectin tissue residue concentrations (ng/g) at 14 days post-administration (0.2 mg/kg, subcutaneous) to parasitized (gastrointestinal nematodes) and non-parasitized lambs. The inserted table shows the estimated withdrawal times for liver and fat in both experimental groups, considering the residue profiles at four post-treatment times. Gastrointestinal parasitism significantly reduced doramectin tissue distribution, resulting in shorter WPs in parasitized (P) than healthy lambs (NP). * *p* < 0.05. Data adapted from Perez et al., 2008 [153].

In contrast, after SC administration, ivermectin and doramectin plasma exposures were lower in steers than in heifers [149]. The reduced plasma drug persistence has been related to the smaller fat depot in the animals with poor body condition. In fact, after SC administration, ivermectin plasma exposure in pigs fed a restrictive diet was reduced in comparison to those fed a normal grower ration diet [150]. Similarly, the longest ivermectin plasma persistence was found in sheep with the highest body weight [151]. In addition, the disease could also affect the disposition of the drug in plasma and, consequently, the tissue residue profiles. The results differ according to the drug considered. For the benzimidazole anthelmintic albendazole, the metabolite plasma profiles in parasitized sheep were higher than those in healthy sheep [152]. As shown in Figure 3, the opposite relation was described for doramectin in lambs, since gastrointestinal parasitism significantly reduced its tissue distribution, resulting in WPs shorter in parasitized than healthy lambs [153].

Concerning the tissue sample to be analyzed to measure drug residues in a specific commodity, it is important to note that the anatomical location of the sample could influence the quantified residue level. For example, enrofloxacin residue concentrations in treated chickens were higher in breast versus thigh [154]. The highest residue level was measured in the intercostal muscles when ivermectin was administered to sheep by the SC route. However, after topical doramectin administration to cattle, the highest residues were quantified in the diaphragm. Both molecules are high lipophilic macrocyclic lactones extensively distributed from the bloodstream to different tissues. The residual ivermectin pattern in muscle could be explained by higher distribution in the infiltrated fat of the intercostal muscles. However, it did not explain the results obtained for doramectin, the diaphragm being a less fatty muscle. Therefore, other factors such as more or less muscle irrigation could be implicated in the different distribution among muscles [155].

### 5.3. Impact of Cooking on the Drug Residue Levels

Most published information about veterinary drug residues is related to their concentrations in raw tissue [156]. However, animal food undergoes further processing before consumption to increase digestibility, sensory properties, and shelf-life [157]. As most types of food are cooked before consumption [158], more information about the cooking effect on residues is required to give a more accurate estimation of consumer exposure to these chemicals. Consequently, a scientific gap of knowledge needs to be addressed.

According to the chemical properties of the molecule, its stability against heating during cooking could change. Table 2 shows the mean drug reductions or increments obtained after commonly used cooking procedures for the different edible animal tissues and the literal conclusion reached by the different researchers. Data on the subject, including the main antibiotics and anthelmintics used in different productions, are reported.

Regarding molecule stability, there are certain discrepancies among researchers. Considering tetracyclines and sulfonamides, all authors agree that these compounds are unstable molecules. However, while some of them indicated that heat treatments are effective in degrading tetracycline and sulfonamide residues to a safe level [157,159,160], others observed that cooking does not guarantee the full breakdown of these compounds [161,162,163,164,165]. Regarding quinolones, although some authors argued that enrofloxacin was an unstable molecule [166], most researchers agreed that it was stable after different cooking methods [167,168]. An increase in enrofloxacin residues after grilling or roasting has even been reported due to the loss of moisture during cooking. This effect led to a higher apparent concentration of the quinolone residue [167]. In contrast, its metabolite ciprofloxacin became an unstable compound [169,170]. Both macrolide [171] and penicillin [172] residues were unstable molecules after the different heat treatments. Rose et al. [173] and Cooper et al. [174] reported that ivermectin was stable during cooking but other researchers indicated that it was an unstable molecule [175]. There also are controversial results concerning the stability of different benzimidazole compound residues. Some authors determined that fenbendazole residues were resistant to degradation under conventional cooking methods [173]; others argued that fenbendazole and oxfendazole concentrations were unstable compounds [176]. There is no clear and consistent explanation for the effect of cooking on oxfendazole/metabolite residue in food. In some cases, it is not known why the variations between replicates were high. Oxfendazole and its metabolite residue concentrations increased in some samples and decreased in others. The lack of homogeneity in the distribution of drug residues was partly associated with the variations found.

There is no fixed stability pattern for molecules. While some of them tend to be heat stable (quinolones, nitroimidazoles, nitrofurans, and anthelmintics), others tend to be more heat labile (tetracyclines, macrolides, sulfonamides, ß-lactams, and aminoglycosides). In addition, some authors reported large variations for the same molecule between the different heat treatments. These variations could be explained both by the dehydration of the food during cooking and by the lack of homogeneity in the distribution of the residue in the tissue. The stability parameter would indicate whether surveillance data obtained by measurements of molecules in raw tissue could be directly applicable for use in consumer exposure and dietary intake calculations or not.

## 6. Antimicrobial Use in Food-Producing Animals: Risk of Residues in Food 

### 6.1. Trends in the Use of Antibacterial Agents in Livestock

Antibacterial administration to food-producing animals has different purposes: therapeutic use for treating an infectious disease caused by bacteria, metaphylactic use for treating a group of animals when only some animals present symptoms of the disease, prophylactic use when treatment is used as a preventive measure ranging from the so-called “subtherapeutic concentrations” to total therapeutic doses, and growth promoter use based on the use of low doses of antimicrobials in feed or water for an extended period to improve growth and production efficiencies.

Despite the prohibition as growth promoters and the tendency to decrease their use, antibiotics are still used in large quantities with this aim in many countries [177]. The global average annual consumption of antimicrobials per kilogram of animal produced was estimated to be greater than 100 mg/kg [178]. In particular, for the main food-producing species, the global average annual consumption of antimicrobials per kilogram of animal produced was about 45, 148, and 172 mg/kg for cattle, chicken, and pig, respectively. This is aggravated by the estimation that this consumption would grow significantly until 2030 [179]. The situation is complex due to the undesirable consequences of the excessive use of antibiotics, mainly antimicrobial resistance (AMR). 

It is even more difficult because globally there are two sides to the same coin: some countries aim to reduce antibiotic use while others rely on it and may even increase it. In 2020, the EU adopted the “Farm to Fork Strategy”, a tool to help shape the EU’s path towards sustainable food systems. There is an urgent need to reduce dependency on pesticides and antimicrobials, reduce excess fertilization, increase organic farming, improve animal welfare, and reverse biodiversity loss. A 50% reduction, by 2030, in antimicrobial sales for farmed animals and aquaculture has been proposed. This critical goal will be supported by regulation implementations (Regulation EU 2019/6 on Veterinary Medicinal Products, Regulation EU 2019/4 on Medicated Feed), which provide a wide range of measures to fight AMR and promote more prudent and responsible use of antimicrobials in animals [10]. However, it is important to note that the starting point to reach this objective is not the same in all European countries. There is a significant disparity between countries in the antibiotic quantities used. The tenth European Surveillance of Veterinary Antimicrobial Consumption (ESVAC) reported data on the sales of veterinary antimicrobial agents from 31 European countries in 2018 [180]. In this ESVAC, the best-selling antibiotics in all countries were tetracyclines (30.7%), penicillins (28.8%), and sulfonamides (8.4%), representing 67.9% of total sales. For the other antimicrobial classes, 0.1% corresponded to first- and second-generation cephalosporins, 0.2% to third- and fourth-generation cephalosporins, 1.9% to amphenicols, and 0.3% to other quinolones. Differences in sales were observed between countries. Expressed in absolute amounts, the antibiotics sold range from 0.6 to 1724 tons. When the amount of antibiotics is normalized, adjusted with the biomass of the livestock population, it is expressed in mg of antibiotics used per PCU (Population Correction Unit; 1 PCU = 1 kg of biomass livestock). The use range varies widely between Nordic countries, such as Norway (2.9 mg/PCU), and some southern countries such as Spain, Italy (about 200 mg/PCU), and Cyprus (466 mg/PCU). These differences were partially associated with differences in bacterial diseases, the composition of the animal population, and the production systems [180]. Fortunately, an overall decrease of 34.6% in sales (mg/PCU) was detected from 2011 to 2018. A decrease in sales of all antimicrobial classes has been observed except for aminoglycosides, amphenicols, lincosamides, and other antibacterials (classified as such in the ATCvet system). The three best-selling antimicrobial classes, tetracyclines, penicillins, and sulfonamides, have decreased by 46%, 14%, and 52%, respectively [180].

On the other hand, there are low- and middle-income countries with a different socioeconomic context, where the need for increased meat production translates into changes in production practices. Therefore, extensive farming systems will be replaced by large-scale intensive farming operations, in which antimicrobials are routinely used. In these countries (Brazil, Russia, India, China, and South Africa), the use of antibiotics will not decrease; on the contrary, a 99% increase in antimicrobial consumption by 2030 was predicted [179]. Most antibacterial usage is aimed at disease prevention, and their use has become an integral part of modern industrialized food-animal production, to the extent that nearly all feed for growing animals is supplemented with antimicrobials in various doses, ranging from the so-called "subtherapeutic concentrations" to full therapeutic doses [181]. Antimicrobial use in pig and poultry production is predicted to double in line with increasing global meat consumption [179]. Unlike previously reported, antimicrobial consumption in animal production contexts of low- and middle-income countries remains mainly undocumented, limiting the ability to establish and monitor progress toward achieving consumption targets [181]. 

The use or non-use of antimicrobials is controversial. Some risks have been associated with not using these substances in animal production, therefore assessing the risk–benefit ratio has been proposed before antibiotic treatment. The prohibition of antibiotic use in animal production has even been viewed as a non-realistic approach [182]. Among the factors that explain the limited effectiveness of the restriction on antibiotics, the most challenging is that of ecological origin [183]. When a resistant population has replaced a population of susceptible wild bacteria in the environment at no cost to adaptation, the resistant population can become very stable in its ecosystem [184]. On the other hand, a large number of studies demonstrated a consistent decrease in the prevalence of antimicrobial resistance in bacteria isolated from food-producing animals or humans following restrictions on the use of medically important antimicrobials in food-producing animals [53].

**Table 2:** Veterinary drug residue stability in different animal tissues cooked by common heat treatments reported in the literature.

Molecule	Matrix	Processing Methods	Mean drug Reductions (−)/Increments (+) Obtained after Cooking	Stability	References
Oxytetracycline (OTC)	Chicken thigh, chest, liver, and meat	Boiling (5 m–100 °C) Microwaving (3 m–900 W) Roasting (30 m–200 °C) Grilling (2.5 m–8 kW)	−56% −70% −63% −25%	OTC residues can be significantly reduced by heat treatments	[166]
	Chicken muscle	Boling (45 m–80 °C)	−47%	Cooking does not guarantee full breakdown of OTC	[162]
	Chicken muscle	Boiling Grilling Frying	−85% −97% −94%	Cooking methods have positive effects on OTC residues in total and partial degradation	[172]
	Bird muscle and liver	Boiling (30 m–100 °C) Microwaving (3 m–full power) Roasting (30 m–200 °C)	−61 −80 −71	Cooking methods can generally reduce OTC con centration in meat	[185]
Doxicycline (DOC)	Chicken muscle, liver and gizzard	Boiling (9/24/85 m–100 °C) Roasting (25/40/60 m–200 °C) Microwaving (3 m–full power)	−79% 75% −88%	Cooking processes do not guarantee full breakdown of these drugs	[163]
	Chicken thigh and breast	Boiling (20 m/30 m/40 m–100 °C) Microwaving (10 m/15 m/20 m–full power) Roasting (40/60/80 m–180 °C)	−28% −33% −28%	DOC is an unstable drug that will be degraded during cooking	[186]
	Pig muscle	Boiling (3/6/9 m–100 °C) Deep-frying (3/6/9 m–170 °C) Microwaving (0.5/0.75/1 m-full power)	−36% −36% −28%	DOC residues are significantly affected by cooking	[157]
	Egg	Boiling (0.5/2/4/6/8 m–100 °C) Frying (0.5/2/4/6 m–180 °C) Microwaving (0.5/1/2/4 m–full power)	−21% −31% −38%	Ordinary cooking does not eliminate all DOC residues present in eggs	[164]
Tetracycline (TC) Chlortetracycline (CTC)	Chicken breast and thigh	Boiling (20 m/30 m/40 m–100 °C) Microwaving (10 m/15 m/20 m– full power) Roasting (40/60/80 m–180 °C)	TC: −52% CTC: −46% TC: −61% CTC: −60% TC: −69% CTC: −62%	TC and CTC are unstable drugs that will be degraded during cooking	[186]
	Pig muscle	Boiling (3/6/9 m–100 °C) Deep-frying (3/6/9 m–170 °C) Microwaving (0.5/0.75/1 m–full power)	TC: −44% CTC: −55% TC: −43% CTC: −55% TC: −32% CTC: −40%	TC and CTC residues are significantly affected by cooking	[157]
	Eggs	Boiling (5/10/15 m–100 °C) Frying (1/3/5 m–160 °C)	CTC: −41% CTC: −80%	CTC residues were highly sensitive to boiling or frying	[187]
Sulfamethazine	Chicken muscle	Deep frying (3/6/9 m–170/180/190 °C)	−27%	Deep-frying ensures safety of sulfamethazine residues consumption in food	[160]
	Piglet muscle	Boiling (5/10/15 m–100 °C) Microwaving (0.5/1/1.5 m–full power) Autoclaving (10/15/20 m–121 °C)	−16% −19% −30%	Heat-treatments do not guarantee full removal of sulfamethazine residues	[165]
Sulfadiazine (SDZ) Sulfamethoxazole (SMX) Sulfaquinoxaline (SQ)	Chicken muscle	Deep frying (3/6/9 m–170/180/190 °C)	SDZ: −37% SMX: −40% SQ: −27%	Deep frying ensures the safety of SDZ, SMX, and SQ residue consumption in food	[160]
	Chicken muscle	Roasting (3/6/9/12 m-170 °C) Microwaving (0.25/0.5/0.75/1 m-full power) Boiling (3/6/9/12 m-100 °C)	SDZ: −3% SMX: −21% SQ: −24% SDZ: −28% SMX: −27% SQ: −34% SDZ: −53% SMX: −44% SQ: −39%	Cooking methods reduce SDZ, SMX, and SQ residues in chicken muscle effectively	[188]
Sulfanilamide	Eggs	Boiling (5/10/15 m-100 °C) Frying (1/3/5 m–160 °C)	−66% −78%	Sulfanilamide residues were highly sensitive to boiling or frying	[187]
Tylosin	Chicken muscle	Boiling (10/20/30 m-100 °C) Microwaving (1/1.5/2 m-full power)	−75% −20%	Exposure to tylosin residues may be reduced with a suitable cooking method	[171]
Timicosin	Chicken muscle	Boiling (30 m-100 °C) Microwaving (15 m-900 W) Frying (10 m–200 °C)	−36% −74% −46%	Sufficient heating temperature and time can reduce nearly 50% of tilmicosin residues	[189]
Enrofloxacin (EFX)	Chicken muscle	Microwaving (3.5 m–full power) Roasting (10 m–200 °C) Boiling (10 m–100 °C) Grilling (10m) Frying (10 m)	−58% +92% −52% +59% −41%	Cooking procedures did not affect quinolone residual levels	[167]
	Chicken thigh, chest, liver, and muscle	Boiling (5 m–100 °C) Microwaving (3 m–900 W) Roasting (30 m–200 °C) Grilling (2.5 m–8 kW)	−60% −52% −64% −34%	EFX residues can be significantly reduced by the application of heat treatments	[166]
Ciprofloxacin (CFX)	Chicken meat	Boiling (5/10 m–100 °C) Deep frying (3/6 m–170 °C) Microwaving (1/2 m–full power)	−29% −34% −52%	Cooking processes can cause a significant decrease in the level of CFX in meat	[170]
	Chicken thigh, chest, liver, and muscle	Boiling (5 m–100 °C) Microwaving (3 m–900 W) Roasting (30 m–200 °C) Grilling (2.5 m–8 kW)	−60% −52% −62% −25%	CFX residues can be significantly reduced by the application of heat treatments	[166]
Gentamicin	Chicken muscle	Boiling (30 m–100 °C) Microwaving (15 m–900 W) Frying (10 m–200 °C)	−36% −50% −56%	Sufficient heating temperature and time can reduce nearly 50% of gentamicin residues	[189]
	Egg	Boiling (1.5/5 m–100 °C) Frying	0% −10%	Gentamicin residue levels in eggs were not reduced by different cooking procedures	[190]
Ampicillin	Chicken muscle	BoilingGrilling Frying	−81%, −94% −90%	Application of different cooking methods has a positive effect on degrading ampicillin residues	[172]
Amoxicillin	Egg	Boiling (5/30/45 m–100 °C) Microwaving (0.5/1/1.5 m–full power) Omelette making (1/2/3 m–130 °C)	−49% −61% −75%	AMX reduction was observed during the cooking procedures	[168]
Nitrofurans: 3-amino-2-oxazolidinone (AOZ) 3-amino-5-morpholinomethyl-2-oxazolidone (AMOZ) 1-aminohydantoin (AHD) Semicarbazide (SEM)	Pig muscle and liver	Frying (5/6 min–medium heat) Grilling (8 m–medium heat) Roasting (20 m–170 °C) Microwaving (2.5 m–800 W)	AOZ: −21% AMOZ: −11% AHD: −17% SEM: −6% AOZ: −10% AMOZ: −7% AHD: +4% SEM: −14% AOZ: −22% AMOZ: −20% AHD: −11% SEM: −17% AOZ: −15% AMOZ: −14% AHD: +1% SEM: −13%	The various stability data presented here demonstrate that AOZ, AMOZ, AHD, and SEM show remarkable chemical stability. They are resistant to conventional domestic cooking procedures	[191]
Ivermectin	Beef muscle	Boiling (9 m–100 °C) Frying (10/13/16 m-177/192 °C)	−45% −48%	Ordinary cooking procedures appear to give an additional safety margin in the exposure to ivermectin residues	[175]
	Pig muscle and liver Cattle muscle	Boiling (20 m–100 °C) Microwaving (full power) Frying	+10% −5% −2%	Ivermectin was found to be stable to the effects of cooking	[173]
	Cattle muscle and liver	Roasting (40 m–190 °C) Frying (12/15 m-high/medium heat)	0% −18%	Ivermectin residues in food are resistant to degradation under conventional cooking	[174]
Levamisole	Beef and pork muscle	Boiling (40 m–100 °C) Roasting (45 m–180 °C) Grilling (9 m-medium heat) Frying (23.5 m) Microwaving (4.5 m–full power)	−6% 0% −11% +13% −7%	Levamisole was stable under normal cooking conditions	[192]
	Cattle muscle and liver	Roasting (40 m–90 °C) Frying (12/15 m–high/medium heat)	+1% −26%	Levamisole residues in food are resistant to degradation under conventional cooking	[174]
Oxfendazole (OFX) Fenbendazole (FEN)	Cattle muscle and liver	Frying Braising (18 m–low heat) Microwaving (full power)	OFX: −77% FEN: +112% OFX: −42% FEN: +10% OFX: −36% FEN: −54%	Oxfendazole concentrations in raw tissue may not be directly applicable for use in consumer exposure	[176]
	Cattle muscle and liver	Roasting (40 m–190 °C) Frying (12/15 m–high/medium heat)	FEN: −5% FEN: −4%	FEN residues are resistant to degradation under conventional cooking	[174]

### 6.2. Residues in Food and Antimicrobial Resistance (AMR)

AMR is the ability of a microorganism (bacteria, viruses, and certain parasites) to prevent an antimicrobial (antibiotics, antivirals, and antimalarials) from working against it [193]. Bacteria evolve in response to the use of antibiotics both in humans and animals. Those bacteria resistant to antibiotics prosper, while antibiotics kill the non-resistant bacteria [194]. 

The antimicrobial arsenal available in livestock, with few exceptions, is very similar to that available in human medicine. As mentioned, most antibiotic use occurs in the farming sector [195]. Antibiotic use in livestock has been linked to the emergence of resistance [196,197], and the transfer of resistant bacteria has been observed in poultry and pig farms among workers, animals, and the environment [198,199,200]. In Veterinary Medicine, AMR has been cataloged into three types: 1. AMR for specific animal pathogens; 2. AMR for zoonotic pathogens; and 3. AMR of the commensal bacteria harbored by animals. The hazards associated with organisms of the gastrointestinal tract and possibly the skin (Type 3) are much more severe ecologically since their biomasses are much greater than those of the other groups [183]. However, animal medicine for food production differs from the critical situation of human medicine because, in livestock, there are no life-threatening infections of multidrug-resistant microorganisms that cause sepsis or chronic conditions that urgently require antibiotic therapy [183]. 

The main concern is that the inappropriate use of antibiotics in food-producing animals can create resistance to antibiotics in non-pathogenic bacteria, whose resistance genes can be transferred to pathogenic bacteria and whose infections are difficult to treat in humans. Due to ciprofloxacin subtherapeutic use in chickens, *E. coli* is resistant to ciprofloxacin, and a similar situation has been found in humans. It points to poultry as the source of the AMR bacteria instead of medical use of the drugs in humans [199]. Consequently, not all antibiotics can be used freely in food-producing animals. The FDA approved ciprofloxacin use in poultry in 1995. Ten years later, the FDA banned its use because nearly 30% of *E. coli* found in chicken breasts were ciprofloxacin resistant. Currently, no fluoroquinolones are approved for use in poultry in the USA, even in an extra-label manner [49].

In the same way, in the EU, some antibiotics have been restricted in food-production animals. The EMA Antimicrobial Advice ad hoc Expert Group (AMEG), considering the WHO categorization, changed the previous antibiotic classification into four different categories, from A to D [201]. Category A (“Avoid”) includes antibiotics not authorized in veterinary medicine but authorized in human medicine. Category B (“Restrict”) includes fluoroquinolones, other quinolones, third- and fourth-generation cephalosporins, and polymyxins. The risk to public health resulting from veterinary use is estimated to be higher than others. These drugs should only be used when there are no alternative antibiotics in a lower category and based on antibiotic susceptibility testing. Category C (“Caution”) includes aminoglycosides, aminopenicillins in combination with beta-lactamase inhibitors, amphenicols, first- and second-generation cephalosporins, macrolides, lincosamides, pleuromutilins, and rifamycins. For these antibiotics, in general, there are alternatives in human medicine, but there are few alternatives in veterinary medicine. These antibiotics should only be used when there is no option in Category D. Category D (“Prudence”) is the lowest risk category. The risk to public health associated with the use of these antibiotics in veterinary medicine is considered low. It includes aminopenicillins (without beta-lactamase inhibitors), beta-lactamase-resistant penicillins, natural narrow-spectrum penicillins, sulfonamides, dihydrofolate reductase inhibitors and combinations, tetracyclines, bacitracin, and spectinomycin. There are no specific recommendations to avoid the use of these antibiotics. However, the general caveat is that responsible use should be followed in daily practice.

Transmission of resistance from animals to humans can take place through a variety of routes. Bacteria and antibiotic residues from food-animal production are spread widely in the environment, mainly with manure, affecting bacteria in both environment and wild fauna. In food-producing animals, the most convenient route of antibiotic administration is oral. As mentioned above, the antibiotic classes most commonly used in food-producing animals are tetracyclines, beta-lactams, and sulphonamides. It was reported [183] that tetracyclines have very low oral bioavailability both in pigs (5–15%) [202,203] and poultry (≤5%) [204]. The unabsorbed antibiotic is exposed to the bacterial population in the gastrointestinal tract [205], being microbiologically active in the feces excreted to the environment. AMR to tetracyclines is commonly associated with multidrug-resistant bacteria, capable of co-selecting genes that confer resistance to critical antibiotics for humans [206]. However, its use is not legally restricted in food-producing animals [183]. Similarly, the low ampicillin bioavailability (10%) after a single dose in pigs led to fast alteration of the intestinal microflora [207].

Furthermore, it is essential to highlight antibiotic use in fish farming, mainly in the salmon industry. Worldwide, over two million metric tons of fish are produced each year. Although the main producing countries are Norway and Chile, consumption reaches the EU, USA, Japan, and increasingly East and Southeast Asia [208]. Without complying with the withdrawal periods, the inappropriate use of antibiotics can lead to high residue levels in fisheries and the environment [209]. Approximately 80% of the antimicrobials used in aquaculture were estimated to enter the environment with their activity mostly intact [210]. Consequently, the environment and wild fauna can be reservoirs of resistance and reintroduce resistant bacteria into the food-animal and human reservoirs. The induced resistance impacts health, resulting in more severe infections that would not have occurred, increased treatment failures, and even death in some cases [53]. Therefore, the digestive tract of a healthy or sick person was described as an open door to the determinants of AMR from various sources, food-producing animals, and the terrestrial and aquatic environments, depending on the risk factors of the individual [183]. 

High levels of multiple AMR have been observed against many microbial organisms affecting humans and animals [211,212,213,214,215]. Carbapenems are not used in food-producing animals; however, genes for resistance to carbapenems have been found in livestock, specifically in pigs and chickens [216,217]. In contrast, colistin is mainly used in food-producing animals, but AMR was detected in livestock and humans [218,219]. Multidrug AMR and potentially pathogenic bacteria have been isolated in seafood from different countries [220,221,222,223,224].

Antibiotic residues in foods of animal origin are less of a concern for direct public health effects than the AMR they generate [225]. Even low concentrations of antibiotics can select for resistant bacteria of animal origin and spread to humans through the environment, food products, and agricultural workers [226]. In such a way, the ingestion of low levels of antibiotic residues in food (meat, milk, eggs, honey, fish) would promote the selection of resistant bacteria [227]. The high level of antibiotic residues found in tissues was associated with high AMR levels. All antibiotic residues tested in food were routinely used in humans and food-producing animals [219], which constitutes a food-borne AMR problem in humans [228]. Moreover, the high exposure to drug residues in food (muscle, liver, kidney, and milk) was considered a risk to human health [229]. 

Given the seriousness of the problem, in 2016, countries reaffirmed their commitment to developing national action plans on AMR, based on the Global Action Plan on Antimicrobial Resistance. It implies following the Global Action Plan objectives, including surveillance to understand the full scale of the problem and mechanisms to stop the misuse of antimicrobial medicines in human health, animal health, and agriculture. The need for stronger systems to monitor drug-resistant infections and the volume of antimicrobials used in humans, animals, and crops and increased international cooperation and funding was emphasized. The countries pledged to foster regulation of antimicrobials, improve knowledge and awareness, promote better practices, and foster innovative approaches using alternatives to antimicrobials and new technologies for diagnosis and vaccines [230].

## 7. Conclusions

The management of drug residues in foods of animal origin is closely linked to rational pharmacotherapy in livestock animals. Drugs of veterinary use must be registered by government agencies, which are also responsible for establishing MRLs for the chemical agents allowed in food. It should be noted that drug residue monitoring plans in food are crucial to check if the rules are being followed.

When the established rules for the control of drug residues are not followed, there is no contribution to the “One Health” paradigm. Various and different human, animal, and environmental adverse health impacts stem from the presence of drug residual concentrations above the permitted levels. The most important adverse effects are those on consumer health, particularly the contribution to antimicrobial resistance. Altogether, the drug residues issue has a relevant global economic impact, which includes the risk of food trade blockage and its many related consequences. 

Therefore, it is crucial that all the actors related to the livestock production chain, including feed producers, farmers, livestock operators, drug manufacturers, veterinary drug sellers, veterinarians, slaughterhouse personnel, etc., assume full responsibility for the prudent use of drugs in food-producing animals, reducing the risk of the presence of non-permitted residue levels in food. Education and awareness campaigns are necessary to inform the risk of the misuse of drug treatments in animals whose tissues are aimed at human consumption. The relationship between drug rational use and avoidance of non-permitted residues in food of animal origin is well established. We should all contribute to avoiding the risky and adverse consequences of the presence of chemical residues in food. For this goal to be fulfilled, there is an obvious need for regulations for the management of veterinary drugs at all levels. In general, these regulations are already established, but further strict controls by relevant authorities are necessary to ensure equivalent compliance in countries from different regions of the world. Overall, this review article contributes to the assessment of different aspects related to drug residues in edible tissues for human consumption, which is heavily dependent on rational pharmacotherapy in food-producing animals.

## Figures and Tables

**Table 1:** Agencies or official bodies that report toxicological evaluations and parameters, such as the no-observed-adverse-effect level (NOAEL), the acceptable daily intake (IDA), the maximum residue limits (MRL), or tolerances established for veterinary drugs.

Official Agencies/Bodies	Area of Application	Links
Codex Alimentarius The Codex Alimentarius Commission (**CAC**) is the body responsible for all matters regarding the implementation of the Joint FAO/WHO Food Standards Programme.	International	http://www.fao.org/fao-who-codexalimentarius/sh-proxy/en/?lnk=1&url=https%253A%252F%252Fworkspace.fao.org%252Fsites%252Fcodex%252FStandards%252FCXM%2B2%252FMRL2e.pdf (accessed on 30 September 2021) http://www.codexalimentarius.org/standards/vetdrugs/en/(accessed on 30 September 2021)
European Medicines Agency (EMA) EMA is a decentralized agency of the European Union (EU) responsible for the scientific evaluation, supervision, and safety monitoring of medicines.	EU	• http://www.ema.europa.eu/ema/index.jsp?curl=pages/medicines/landing/vet_mrl_search.jsp&mid=WC0b01ac058006488e (accessed on 30 September 2021)
The Food and Drug Administration (FDA) FDA is an agency within the U.S. Department of Health and Human Services. It consists of the Office of the Commissioner and four directorates overseeing the core functions of the agency: Medical Products and Tobacco, Foods and Veterinary Medicine, Global Regulatory Operations and Policy, and Operations.	USA	• https://www.accessdata.fda.gov/scripts/cdrh/cfdocs/cfcfr/CFRSearch.cfm?CFRPart=556&showFR=1 (accessed on 30 September 2021)
Government of Canada Health Canada through the Veterinary Drugs Directorate (VDD) evaluates and monitors the safety, quality and effectiveness, sets standards, and promotes the prudent use of veterinary drugs administered to food-producing and companion animals.	Canada	https://www.canada.ca/en/health-canada/services/drugs-health-products/veterinary-drugs/maximum-residue-limits-mrls/list-maximum-residue-limits-mrls-veterinary-drugs-foods.html (accessed on 30 September 2021) https://www.canada.ca/en/health-canada/services/drugs-health-products/veterinary-drugs.html (accessed on 27 September 2021)
Australian Government Department of Agriculture, Water and the Environment. Federal Register of Legislation	Australia and New Zealand	https://www.legislation.gov.au/Details/F2015L00468 (accessed on 27 September 2021) https://www.agriculture.gov.au/ag-farm-food/food/nrs/databases (accessed on 30 September 2021)

## Data Availability

Not applicable.
